# The Level of Awareness Among Surgical Physicians Regarding Surgical Site Infections and the Risks Associated With Wound Infections in Makkah

**DOI:** 10.7759/cureus.51111

**Published:** 2023-12-26

**Authors:** Ahmad S Maoudah, Lujain Alshareef, Raed M Babukur, Abdulrahman Alharthi, Bader Y Alnashri, Nasser Al Shanbari, Fayez A Alwadani, Alhassan Almaghrabi

**Affiliations:** 1 Department of Medicine and Surgery, College of Medicine, Umm Al-Qura University, Makkah, SAU; 2 Department of Medicine and Surgery, College of Medicine, Ibn Sina National College, Jeddah, SAU; 3 Department of General Surgery, Al-Noor Specialist Hospital, Makkah, SAU

**Keywords:** surgical site infections (ssi), physicians, makkah, knowledge, infection, awareness

## Abstract

Background and aims: Surgical site infections (SSIs) are a significant contributor to mortality rates globally; therefore, to avoid these lethal complications, it is critical to incorporate patient safety and high-quality treatment approaches. This study aims to assess surgical physicians' awareness of SSIs and risks of wound infections in Makkah City, Saudi Arabia.

Methods: A descriptive cross-sectional study was performed among surgical physicians and interns in Makkah city hospitals through an online questionnaire from February 2023 to March 2023.

Results: 122 surgical physicians were enrolled in the study. The age of the majority of participants was between 20 and 30 (52.5%). According to the data, 55.7% of respondents had fair knowledge.

Conclusion: Only 4.1% of physicians had a good level of knowledge. Thus, we recommend Makkah hospitals offer academic sessions to surgical physicians about preventive measures for high-quality care of SSIs in order to raise their levels of awareness and knowledge.

## Introduction

A surgical site infection, or SSI, is a potentially severe consequence of surgery that occurs when there is a microbial infection of the surgical wound within one month of the operation or a year later [1]. SSIs are thought to be a significant source of morbidity and mortality worldwide [2]. SSIs are a common and concerning post-operative sequelae and they represent 2% of surgical complications and more than 20% of infections that are related to healthcare [3]. According to a study conducted in the United States of America, the prevalence of SSIs is 1.07% [4]. Moreover, according to a UK survey, 14.5% of individuals had SSIs [5]. The frequency of SSIs is correlated with a greater risk of death, length of hospital readmission stays, and monetary expense, which is influenced by a variety of factors [4]. Furthermore, another study found that SSIs represent a higher cost among healthcare-associated infections (HCAIs), with an estimated yearly financial burden of over $3 billion nationwide [6]. However, SSIs can be avoided or reduced by a variety of cost-effective strategies, such as skin preparation at the surgical site, operative cleaning, dietary support, bathing before surgery, taking antibiotics, removing hair, and mechanical preparation of the bowel [7,8].

While it is simple and affordable to use basic safety precautions and excellent hand hygiene during invasive surgeries, it still necessitates staff training and surveillance systems [9]. As is widely known, preventing SSIs is key to putting the idea of patient safety and great care into practice [9]. The majority of studies in the literature describe how much nurses know and are aware of SSIs [10]. A previous study, which was conducted at King Abdulaziz University Hospital, involved 119 doctors and found that eight doctors (6.7%) had strong knowledge, 75 (63.0%) had fair understanding, and 36 (30.2%) had poor knowledge [7]. For all medical personnel, patient safety is a top priority, and each of them takes part in patient care [11]. Doctors are also involved in patient care and should be evaluated in addition to nurses, who play a significant role in preventing SSIs.

In 2006, a survey was conducted to determine the frequency of HCAIs among patients in UK hospitals. The survey indicated that around 8% of hospitalized patients had an HCAI. Among these infections, SSIs made up 14% of the total. Additionally, it was found that almost 5% of patients who had undergone a surgical procedure developed an SSI. However, it should be noted that the survey likely underestimated the actual prevalence of SSIs because many of these infections occur after the patient has been discharged from the hospital [12]. Therefore, our study aims to determine the level of awareness among surgical physicians regarding SSI and the risks associated with wound infections in Makkah.

## Materials and methods

A descriptive cross-sectional study was carried out between February 7 to March 23, 2023. Participants of this study were all surgical physicians, including consultants, specialists, residents, and medical interns affiliated with different surgical departments (general surgery, orthopedics, pediatric surgery, urology, obstetrics and gynecology, neurosurgery, cardiac surgery) in Makkah. Excluded are physicians who disagreed to participate in the study. Within an estimated population of 454 surgeons in Makkah hospitals [13], 151 surgeons have completed the survey with a 95% confidence interval.

This study was approved by the Umm Al-Qura Institutional Review Board (IRB) with approval number HAPO-02-K-012-2023-02-1439. Informed consent was obtained from all study participants before they completed the questionnaire. Confidentiality and anonymity of the participants were strictly maintained throughout the study.

A convenience sampling method was utilized to select the participants. Data for this study were collected through a self-administered multiple-choice survey that was distributed to the surgical physicians through online apps and in person during their routine work shifts. A validated questionnaire was developed based on a thorough review of the existing literature on SSIs and wound infections [1]. The questionnaire included a series of 20 multiple-choice questions covering various aspects related to SSIs, risk factors, prevention strategies, and management approaches.

Quantitative data collected from the completed surveys were analyzed using RStudio (R version 4.2.2; RStudio: Integrated Development for R. RStudio, PBC, Boston, MA). The sum of all of the right answers was used to determine the total score for knowledge items (n=20), where a correct response was assigned a score of 1 and other responses were assigned a score of 0. Therefore, the knowledge score ranged from 0 to 20. Knowledge categories were assigned as the following: good knowledge (participants with ≥80% right answers), acceptable understanding (participants who answered correctly between 50% and 79%), and inadequate understanding (participants with <50% right answers). Frequencies and percentages were used to express the categorical data, while continuous data (knowledge scores) were represented by the median and interquartile range (IQR). Knowledge-related factors were evaluated using a Wilcoxon rank-sum test for variables with two categories or a Kruskal-Willi’s rank-sum test for variables with three or more categories. The significantly associated factors were further used as independent variables in multiple generalized linear models to assess the independent predictors of knowledge. Results were presented as beta coefficients and 95th percentiles for confidence intervals (CIs). Statistics were significant when the p-value was less than 0.05.

## Results

Demographic characteristics 

Initially, we received 151 responses from those who agreed to participate on the online platform. However, we excluded 11 responses from those who were working outside Makkah and 18 responses from physicians working in non-surgical specialties. Therefore, we analyzed data from 122 participants in the current study. Almost half of them were between 20 to 30 years of age (52.5%), were single (50.8%), and had a monthly income of >19,000 SAR (45.9%). Most physicians were Saudis (84.4%). Residents represented 42.6% of the sample, whereas interns affiliated with surgical departments, specialists, and consultants represented 21.3%, 14.8%, and 21.3%, respectively (Table 1).

**Table 1:** Demographic characteristics of physicians.

Parameter	Category	N (%)
Age (years)	20-30	64 (52.5%)
	31-40	31 (25.4%)
	41-50	19 (15.6%)
	51-60	8 (6.6%)
Position	Intern	26 (21.3%)
	Resident	52 (42.6%)
	Specialist	18 (14.8%)
	Consultant	26 (21.3%)
Nationality	Non-Saudi	19 (15.6%)
	Saudi	103 (84.4%)
Marital status	Single	62 (50.8%)
	Married	60 (49.2%)
Monthly income (SAR)	< 10,000	22 (18.0%)
	10,000 to 19,000	44 (36.1%)
	> 19,000	56 (45.9%)

Physicians' responses to knowledge items regarding surgical site infections

In general, only 38.5% of physicians correctly defined SSI, and 31.1% of them correctly identified that superficial incisional SSI accounts for more than half of SSI cases. Approximately one-half of physicians could identify SSI classifications (50.8%) and perceived that* Staphylococcus aureus *is one of the most isolated organisms in SSI (50.8%), preoperative showering with antimicrobial soaps is recommended to prevent SSI (50.0%) and that prophylactic antibiotics are discontinued within 24 to 48 hours (49.2%). The majority of physicians correctly agreed that one hour before surgery is the ideal window of time to administer prophylactic antibiotics (77.0%), pre-operative skin cleaning is done to lessen the amount of skin flora (91.8%), steroids could impair wound healing (80.3%), infected wounds can exhibit purulent pus (80.3%) and that fistula formation, increased cost of care and death could all be complications of SSIs (81.1%). About one-third of physicians declared that fidaxomicin is not a common antibiotic for the pre-operative prophylaxis against SSIs (36.1%), having hairy skin is the least associated with SSIs (32.0%), the best time for hair removal is just prior to surgical incision (37.7%) and that clean-contaminated wounds include an entry of a hollow viscus, an incision done under sterile circumstances, and the lack of active infection (38.5%). More details about the responses of physicians to knowledge items are provided in Table 2.

**Table 2:** Physicians' responses to knowledge items regarding surgical site infections. The asterisk indicates the correct response

Parameter	Category	N (%)
The United States Centers for Disease Control and Prevention (CDC) has developed criteria that define SSI as an:	Infection related to an operative procedure that occurs at or near the surgical incision within 14 days of the procedure	51 (41.8%)
	Infection related to an operative procedure that occurs at or near the surgical incision within 30 days of the procedure, or within 1 year if prosthetic material is implanted at surgery*	47 (38.5%)
	Infection related to an operative procedure that occurs at or near the surgical incision within 60 days of the procedure	9 (7.4%)
	Infection related to an operative procedure that occurs at or near the surgical incision within 90 days of the procedure	15 (12.3%)
SSIs are classified into incisional SSIs; which can be superficial; deep or organ/space SSIs. Superficial SSI means:	Infection involving the epidermis and dermis layers only	45 (36.9%)
	Infection involving both only the skin and subcutaneous tissue*	62 (50.8%)
	Infection involving fascial and muscle layers	10 (8.2%)
	Infection involving internal organs manipulated during operation	5 (4.1%)
Which is true about SSI classification?	Superficial incisional SSI occurs within 14 days after operation	56 (45.9%)
	Superficial incisional SSI accounts for more than half of all SSIs*	38 (31.1%)
	Deep organ SSI occurs within 60 days after operation	16 (13.1%)
	Deep incisional SSI is more common than superficial incisional SSI and organ/space SSI	12 (9.8%)
One of the most common isolated organisms in SSI is:	Staphylococcus aureus*	62 (50.8%)
	Streptococcus pyogenes	10 (8.2%)
	Escherichia coli	9 (7.4%)
	A+C.	41 (33.6%)
The best time for administrating prophylactic antibiotics is:	Within 60 minutes prior to surgery*	94 (77.0%)
	Within 90 minutes prior to surgery	12 (9.8%)
	Within 120 minutes prior to surgery	14 (11.5%)
	Within 180 minutes prior to surgery	2 (1.6%)
Chances of developing SSI are:	1-3%*	34 (27.9%)
	3%-5%	46 (37.7%)
	10%-5%	34 (27.9%)
	15%-20%	8 (6.6%)
All the following pre-operative antibiotics are commonly used, except:	Cefazolin	11 (9.0%)
	Cefoxitin	15 (12.3%)
	Vancomycin	52 (42.6%)
	Fidaxomicin*	44 (36.1%)
Which statement is correct about wound classification?	Wound created in herniorrhaphy is considered as a clean-contaminated wound	9 (7.4%)
	Appendiceal abscess is considered as a contaminated wound	18 (14.8%)
	Clean-contaminated wound is defined as an incision under sterile condition; entrance of a hollow viscus with no active infection *	47 (38.5%)
	Bowel obstruction with enterotomy and spillage of contents is considered as a dirty wound	48 (39.3%)
Which one of these risk factors is LEAST associated with SSI?	Prolonged pre-operative stay	31 (25.4%)
	Poor post-operative glycemic control	36 (29.5%)
	Hairy skin*	39 (32.0%)
	Type of wound	16 (13.1%)
Complications of SSI include which of the following	Increased cost of care	8 (6.6%)
	Fistula formation	12 (9.8%)
	Death	3 (2.5%)
	All of the above*	99 (81.1%)
The CDC recommendations for the prevention of SSI include which of the following?	Pre-operative showering with antimicrobial soaps*	61 (50.0%)
	Blood glucose target level of less than 250 mg/dl	37 (30.3%)
	Maintaining mild hypothermia	6 (4.9%)
	Advising patients to shower at least 1 day prior to surgery	18 (14.8%)
Infected wounds can exhibit which one of these presentations?	Sweet smell	1 (0.8%)
	Purulent pus*	98 (80.3%)
	Normothermia	16 (13.1%)
	Painlessness	7 (5.7%)
Prophylactic antibiotics are discontinued after surgery within:	4 to 8 hours	31 (25.4%)
	12 to 18 hours	18 (14.8%)
	24 to 48 hours*	60 (49.2%)
	72 to 96 hours	13 (10.7%)
Regarding hair removal for surgical patients; when is the best time:	Just prior to surgical incision*	46 (37.7%)
	30 minutes prior to surgery.	9 (7.4%)
	2 hours prior to surgery	14 (11.5%)
	The night prior to surgery	53 (43.4%)
Regarding hair removal for surgical patients; it is best done by:	Shaving	57 (46.7%)
	Clipping*	57 (46.7%)
	Waxing	3 (2.5%)
	Electrolysis	5 (4.1%)
Which one of these factors impairs wound healing?	Steroid use*	98 (80.3%)
	Hyperthermia	5 (4.1%)
	Exposure to water	17 (13.9%)
	Protein-rich food	2 (1.6%)
In assessing nutritional status for a surgical patient; which statement is correct?	Serum albumin level is the most commonly used marker to assess nutritional status*	71 (58.2%)
	Serum magnesium is a preferred marker over serum albumin	20 (16.4%)
	Assessing the patient through inspection and further examination should be enough	26 (21.3%)
	Poor nutritional status is not considered as a risk factor for SSI	5 (4.1%)
Based on World Health Organization; the fourth step in hand hygiene technique is:	Rub hands palm to palm	18 (14.8%)
	Rotational rubbing, backwards and forwards with clasped fingers of right hand in left palm and vice versa	43 (35.2%)
	Palm to palm with fingers interlaced*	24 (19.7%)
	Backs of fingers to opposing palms with fingers interlocked	37 (30.3%)
The first step in surgical scrubbing is:	Ensure that your sleeves are at least two to three inches above your elbows	12 (9.8%)
	Remove any watches and rings from your hands*	71 (58.2%)
	Open out your gown pack onto a clean table, only grabbing the outermost edges to maximize the sterile field	36 (29.5%)
	Adjust water flow and temperature	3 (2.5%)
The purpose of pre-operative skin cleansing is:	To achieve good-looking skin	3 (2.5%)
	To reduce the risk of skin cancer	3 (2.5%)
	To achieve a faster operation	4 (3.3%)
	To reduce the burden of skin flora, thus reducing the risk of SSI*	112 (91.8%)

Description of the Knowledge Score and Categories

**Figure 1:**
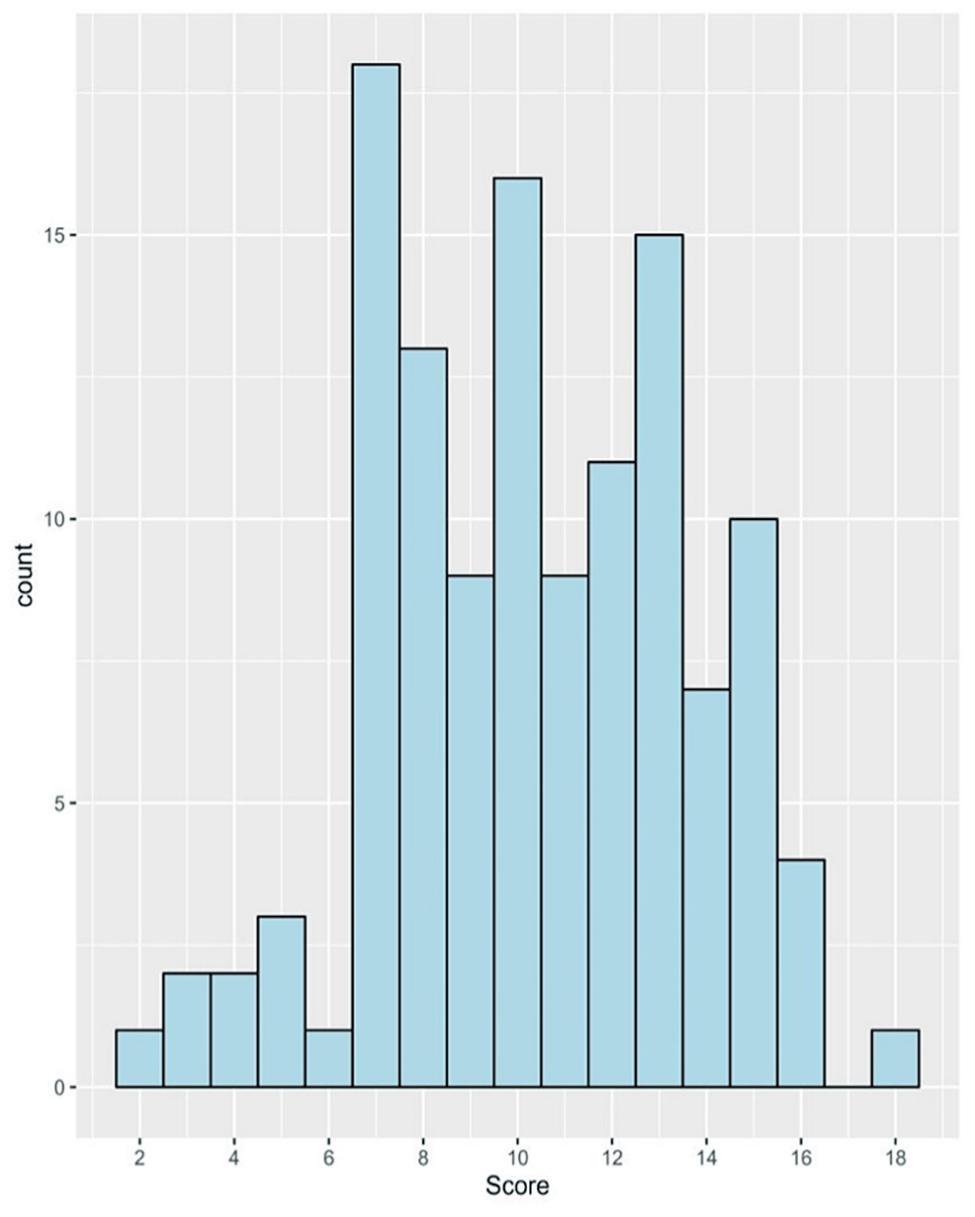
A histogram showing the distribution of the knowledge score among physicians under study.

**Figure 2:**
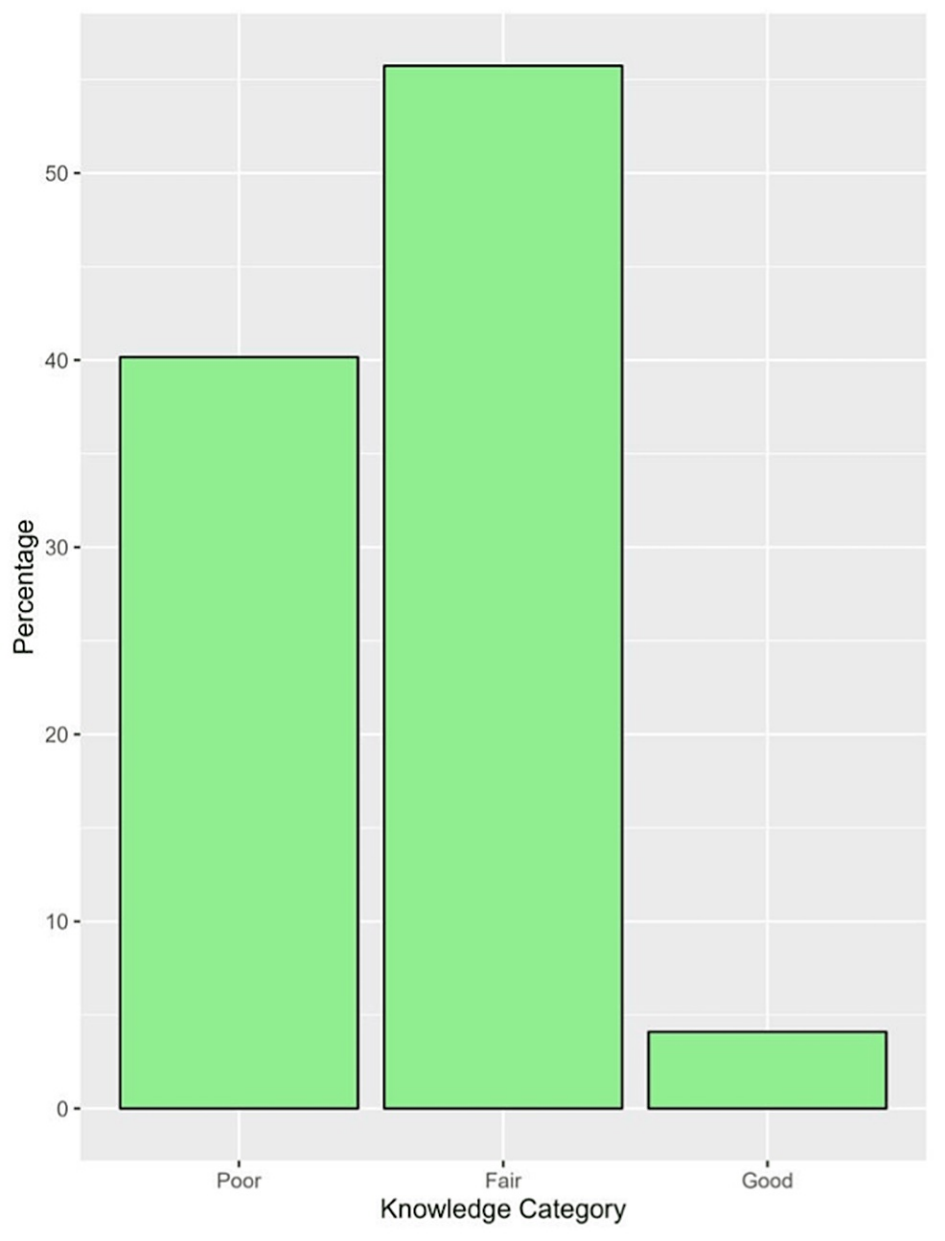
The percent of knowledge categories among physicians under study.

In general, the median (IQR) knowledge score of all physicians was 10 (8 to 13) with a minimum of 2 and a maximum of 18. The distribution of the knowledge score is depicted in Figure 1. Based on knowledge categories, poor knowledge was prevalent among 40.2%, fair knowledge among 55.7%, and good knowledge among 4.1% (Figure 2).

Factors and Predictors of Physicians' Knowledge

Results of the inferential analysis showed significantly higher knowledge scores among consultants (median = 10.0, IQR = 9.0 to 12.8), specialists (median = 12.5, IQR = 10.0 to 13.8), and residents (median = 11.0, IQR = 8.0 to 13.0) compared to interns (median = 8.0, IQR = 7.0 to 10.0, p = 0.004). Additionally, knowledge scores increased consistently with an increased income (median = 9.0, IQR = 7.2 to 10.0 for the < 10,000 SAR group, median = 10.5, IQR = 7.0 to 13.0 for the 10,000 to 19,000 SAR group and median = 11.0, IQR = 9.0 to 13.0 for the > 19,000 SAR group, p = 0.045). However, other demographic characteristics were not significantly associated with physicians' knowledge (Table 3). 

**Table 3:** Factors associated with physicians' knowledge scores.

Parameter	Category	Median (IQR)	p-value
Age (years)	20-30	10.0 (7.0, 13.0)	0.645
	31-40	12.0 (8.0, 13.0)	
	41-50	10.0 (8.5, 11.5)	
	51-60	11.5 (9.8, 13.0)	
Position	Intern	8.0 (7.0, 10.0)	0.004
	Resident	11.0 (8.0, 13.0)	
	Specialist	12.5 (10.0, 13.8)	
	Consultant	10.0 (9.0, 12.8)	
Nationality	Non-Saudi	10.0 (8.5, 12.5)	0.898
	Saudi	10.0 (7.5, 13.0)	
Marital status	Single	10.0 (8.0, 13.0)	0.528
	Married	10.0 (8.0, 13.0)	
Monthly income (SAR)	< 10,000	9.0 (7.2, 10.0)	0.045
	10,000 to 19,000	10.5 (7.0, 13.0)	
	> 19,000	11.0 (9.0, 13.0)	

On the regression analysis, the physician's position was the sole independent predictor of knowledge, where higher knowledge was apparent among residents (beta = 3.6, 95% CI, 1.3 to 5.9, p = 0.002), specialists (beta = 4.1, 95% CI, 1.2 to 7.1, p = 0.007) and consultants (beta = 3.0, 95% CI, 0.1 to 5.9, p = 0.042, Table 4).

**Table 4:** Results of the regression analysis for the predictors of knowledge regarding SSI among physicians.

Parameter	Category	Beta	95% CI	p-value
Position	Intern	Reference	Reference	
	Resident	3.63	1.33, 5.93	0.002
	Specialist	4.14	1.20, 7.09	0.007
	Consultant	3.03	0.14, 5.93	0.042
Monthly income (SAR)	< 10,000	Reference	Reference	
	10,000 to 19,000	-1.31	-3.70, 1.07	0.282
	> 19,000	-1.10	-3.92, 1.72	0.448

## Discussion

The current study aimed to determine the level of awareness among surgeons regarding SSI and the risks associated with wound infections in Makkah. Our results indicate that only 4.1% of surgical physicians were knowledgeable about SSIs, according to our knowledge categories. More than half of the participants scored a level of fair knowledge. The topic of SSI is significant as it represents a cause of increased morbidity and mortality [14], as well as an economic cost to the healthcare system. Thus, there’s a need for well-trained healthcare providers capable of reducing the burden of SSIs via proper practice and sufficient knowledge [9].

The three responses with the highest scores in our questionnaire and a similar study conducted in Hail City were related to the following concepts: First, 87.6% in Hail hospitals identified the purpose of skin cleansing, compared to 91.8% in Makkah hospitals. Second, 79.4% of respondents in Hail hospitals were able to identify the factors impairing wound healing, compared to 80.3% in Makkah hospitals. Finally, 85.6% in Hail hospitals were aware of the presentation of infected wounds, compared to 80.3% in Makkah hospitals. On the other hand, the three responses with the lowest scores in both Hail hospitals and Makkah hospitals were assigned to the following concepts: First, only 25% of physicians were aware of the steps of hand hygiene in Hail hospitals, compared to 19.7% in Makkah hospitals. Second, knowledge regarding the prevalence of SSI was 26.8% in Hail hospitals, compared to 27.9% in Makkah hospitals. Finally, the awareness of SSI classifications was recorded at 63.9% in Hail hospitals, indicating that the participants had fair knowledge, while Makkah hospitals recorded only 31.1%, indicating the participants had poor knowledge [2].

The topic of SSIs has been previously studied by both physicians and nurses. However, our study included only surgical physicians. A separate study, involving both physicians and nurses, conducted in the city of Makkah during the Hajj season of 2019, showed that merely 10% of the participants were able to provide precise definitions of SSIs and demonstrated proficiency by correctly answering over 80% of the associated questions. Similar to our results, this study suggested that the level of experience has a significant role in the knowledge about SSI [1].

Additionally, another study that evaluated the knowledge of medical physicians in King Abdulaziz University Hospital, Jeddah concluded that 6.7% of participating doctors had good knowledge regarding SSIs. Similar to the previous study and our own, the average knowledge score increased with experience [7]. However, according to our results, the previous study did not include consultants, who had lower mean knowledge scores when compared to residents and specialists. We found that lack of experience was the main cause of insufficient knowledge, as seen in interns having the lowest level of both experience and score on our scale. Nonetheless, consultants who may have relied purely on experience were less knowledgeable than residents and specialists. We believe that experience and constant revision are equally essential for optimal performance levels. The participating doctors were most familiar with questions about skin cleansing, factors inhibiting wound healing, and presentations of infected wounds. On the contrary, they were least familiar with questions related to steps of hand hygiene, classification of SSIs, and prevalence of SSIs. This further adds to our earlier observation regarding experience and revision, as participating physicians had the highest scores in questions related to practical information but lacked fundamental knowledge.

While practical experience is crucial for the success of surgeons, it is essential to recognize that the role of theoretical and fundamental knowledge should not be underestimated [15]. This is seen in some of the questions listed above, as well as in the generally low scores of participants. We recommend that clinicians should give more attention to the basics of medical practice. Some of our limitations resulted from the type of study chosen, a cross-sectional survey. So, recall bias is possible and cannot reflect real practice. Moreover, further studies with larger sample sizes would reflect better findings in understanding SSIs.

## Conclusions

To provide better wound care and high-quality patient care, the level of knowledge among medical doctors regarding SSIs and the dangers of wound infections needs to be improved, as only 4.1% of doctors in the current study had a good understanding of SSIs and the risk of wound infections. Thus, we advise Makkah hospitals to increase the number of courses and sessions they offer medical interns, residents, specialists, and consultants to raise their level of awareness and knowledge of SSIs.
